# Chloroplast genes as genetic markers for inferring patterns of change, maternal ancestry and phylogenetic relationships among *Eleusine* species

**DOI:** 10.1093/aobpla/plt056

**Published:** 2013-12-19

**Authors:** Renuka Agrawal, Nitin Agrawal, Rajesh Tandon, Soom Nath Raina

**Affiliations:** 1 Laboratory of Cellular and Molecular Cytogenetics, Department of Botany, University of Delhi, Delhi 110007, India; 2 Cluster Innovation Centre, University of Delhi, Delhi 110007, India; 3 Present address: Amity Institute of Biotechnology, Amity University, Sector 125, Noida 201303, Uttar Pradesh, India

**Keywords:** cpSSR, *Eleusine*, PCR–RFLP, phylogeny, Poaceae, *trn*K gene sequence.

## Abstract

Assessment of phylogenetic relationships is an important component of any successful crop improvement programme, as wild relatives of the crop species often carry agronomically beneficial traits. Since its domestication in East Africa, *Eleusine coracana* (2*n* = 4*x* = 36), a species belonging to the genus *Eleusine* (*x* = 8, 9, 10), has held a prominent place in the semi-arid regions of India, Nepal and Africa. The patterns of variation between the cultivated and wild species reported so far and the interpretations based upon them have been considered primarily in terms of nuclear events. We analysed, for the first time, the phylogenetic relationship between finger millet (*E. coracana*) and its wild relatives by species-specific chloroplast deoxyribonucleic acid (cpDNA) polymerase chain reaction–restriction fragment length polymorphism (PCR–RFLP) and chloroplast simple sequence repeat (cpSSR) markers/sequences. Restriction fragment length polymorphism of the seven amplified chloroplast genes/intergenic spacers (*trn*K, *psb*D, *psa*A, *trn*H–*trn*K, *trn*L–*trn*F, *16*S and *trn*S–*psb*C), nucleotide sequencing of the chloroplast *trn*K gene and chloroplast microsatellite polymorphism were analysed in all nine known species of *Eleusine*. The RFLP of all seven amplified chloroplast genes/intergenic spacers and *trn*K gene sequences in the diploid (2*n* = 16, 18, 20) and allotetraploid (2*n* = 36, 38) species resulted in well-resolved phylogenetic trees with high bootstrap values. *Eleusine coracana*, *E. africana*, *E. tristachya*, *E. indica* and *E. kigeziensis* did not show even a single change in restriction site. *Eleusine intermedia* and *E. floccifolia* were also shown to have identical cpDNA fragment patterns. The cpDNA diversity in *Eleusine multiflora* was found to be more extensive than that of the other eight species. The *trn*K gene sequence data complemented the results obtained by PCR–RFLP. The maternal lineage of all three allotetraploid species (AABB, AADD) was the same, with *E. indica* being the maternal diploid progenitor species. The markers specific to certain species were also identified.

## Abstract (precis)

Phylogenetic relationship between the nine species of *Eleusine* was investigated based on RFLP of the seven amplified chloroplast genes/intergenic spacers, *trn*K gene sequence and cpSSR markers. The maternal genome donor (*E. indica*, 2n=2x=18) of the allotetraploid (2n=4x=36, 2n=2x=38) *Eleusine* species, and the phylogenetic relationships between cultivated *E. coracana* (2n=4x=36) and wild species have been successfully resolved. The species-specific markers were also identified. The explicit identification of the maternal parent and that of the immediate wild progenitor of finger millet will be immensely useful for future genetic improvement and biotechnological program(s) of the crop species.

## Introduction

The wild relatives of crop species often carry beneficial alleles that are effective against various biotic and abiotic stresses. They hold the key to successful crop improvement programmes through introgression of desired genes from wild to cultivated crop species (Dida and Devos 2006). In this context, the assessment of phylogenetic relationships at the inter-specific level and the identification of gene pools are considered important. Based on chloroplast deoxyribonucleic acid (cpDNA) diversity, we obtained information on the molecular phylogeny of finger millet (*Eleusine coracana)* *vis-à-vis* its wild relatives.

The genus *Eleusine* is a member of the tribe Eragrosteae, subfamily Chloridoideae and the family Poaceae. It is a small genus of nine species, which includes six diploid (2*n* = 2*x* = 16, 18, 20) and three polyploid (2*n* = 4*x* = 36, 38) species (Bisht and Mukai 2002; Liu *et al.* 2011). These species are widely distributed in the tropical and subtropical regions of Africa, Asia and South America (Phillips 1972). East Africa is considered to be the centre of diversity for the genus (Bisht and Mukai 2002).

*Eleusine coracana* (2*n* = 4*x* = 36), commonly known as finger millet or ragi, is the only economically important species of the genus. After sorghum and pearl millet, finger millet ranks third in cereal production in the semi-arid regions of the world (Bisht and Mukai 2002). It is widely cultivated in the arid and semi-arid regions of East Africa, India, Nepal and many other Asian countries for its grain and fodder value (Verma 2009). The grain is widely used for preparing bread, cakes, soup, puddings, porridge and fermented beverages (Hilu and deWet 1976; Chandrashekar 2010; Neves 2011). Finger millet is a rich source of essential amino acids and polyphenols, and it has comparatively higher levels of calcium and iron than other known cereals (Barbeau and Hilu 1993; Chandrashekar 2010). It has a number of medicinal properties as well, particularly in controlling blood sugar levels in diabetic patients (Duke and Wain 1981; Chandrashekar 2010; Pradhan *et al.* 2010).

The assessment of genomic relationships between *E. coracana* and its allied species has been a subject of comprehensive investigations at the morphological, cytogenetic, biochemical and DNA level. Chromosome research has demonstrated the significant role of polyploidy and aneuploidy in the evolution of the genus (Chennaveeraiah and Hiremath 1974; Hiremath and Chennaveeraiah 1982; Hiremath and Salimath 1991; Bisht and Mukai 2000, 2001*a*, *b*, 2002). Biochemical, nuclear and cpDNA markers have provided valuable insight into relationships, and on the origin of the crop species (Hilu *et al.* 1978; Hilu 1988, 1995; Hilu and Johnson 1992, 1997; Werth *et al.* 1993, 1994; Salimath *et al.* 1995*a*; Neves *et al.* 2005; Dida *et al.* 2007, 2008; Liu *et al.* 2011). The 2*n* number and the genomic formula proposed on the basis of earlier studies are given in Table 1.

**Table 1.** Plant materials used in the present study. ^a^Dr. Mathews M. Dida, Kenya; USDA, United States Department of Agriculture, USA; ILRI, International Livestock Research Institute, Ethopia; NBPGR, National Bureau of Plant Genetic Resources, India. ^b^Bisht and Mukai (2002).

Species	Accession number	Source^a^	2*n*	Genome^b^ formula	Growth habit
*E. coracana*	PI 482594 PI 462778 PI 462779	USDA USDA USDA	36	AABB	Annual
*E. africana*	EC 541535 EC 541536 PI 226270 PI 315700	Dida, Kenya Dida, Kenya USDA USDA	36	AABB	Annual
*E. tristachya*	PI 477078 PI 331791	USDA USDA	18	AA	Annual
*E. indica*	PI 442480	USDA	18	AA	Annual
*E. floccifolia*	PI 196853	USDA	18	BB	Perennial
*E. multiflora*	PI 226067	USDA	16	CC	Annual
*E. jaegeri*	PI 273888	USDA	20	DD	Perennial
*E. kigeziensis*	1112 1079	ILRI ILRI	38	AADD	Perennial
*E. intermedia*	S. No 116	ILRI	18	AB	Perennial
*D. aegyptium*	IC-285214	NBPGR			

In spite of the enormous amount of information, there still exists considerable disagreement over the identification of the diploid ancestors of the three polyploid species and the level of speciation and evolutionary relationships among the nine species of *Eleusine* (Phillips 1972, 1995; Hilu and Johnson 1997; Lye 1999; Bisht and Mukai 2000, 2001*a*, 2002; Devarumath *et al.* 2005; Neves *et al.* 2005; Liu *et al.* 2011). *Eleusine coracana* (2*n* = 4*x* = 36) (AABB) is considered to be an allotetraploid and has been domesticated from its wild progenitor, *Eleusine africana* (2*n* = 4*x* = 36) (AABB) (Chennaveeraiah and Hiremath 1974; Hilu and deWet 1976; Hilu *et al.* 1978; Hilu 1988, 1995; Hiremath and Salimath 1992; Werth *et al.* 1994; Hilu and Johnson 1997; Bisht and Mukai 2000, 2001*a*, *b*; Devarumath *et al.* 2005; Neves *et al.* 2005; Dida *et al.* 2007, 2008). The genetic maps generated by Dida *et al.* (2007) showed that *E. coracana* and *E. africana* are allotetraploids. There is also strong evidence to suggest that *Eleusine indica* (2*n* = 2*x* = 18) (AA) is the maternal genome donor of *E. coracana* and *E. africana* (Hilu 1988; Hiremath and Salimath 1992; Hilu and Johnson 1997; Bisht and Mukai 2000, 2001*a*; Neves *et al.* 2005). Bisht and Mukai (2000, 2001*a*, 2002) indicated that *Eleusine floccifolia* (2*n* = 2*x* = 18) could be the BB donor species of *E. coracana*. This has, however, been refuted by others (Hiremath and Salimath 1992; Neves *et al.* 2005; Devarumath *et al.* 2010; Liu *et al.* 2011). According to these authors, the BB genome donor species remains unidentified and may possibly be extinct. *Eleusine kigeziensis* (2*n* = 4*x* = 36 or 38) (AADD) is the third tetraploid species of the genus. *Eleusine indica* (2*n* = 2*x* = 18) (AA) and *E. jaegeri* (2*n* = 2*x* = 20) (DD) are proposed to be the wild progenitors of *E. kigeziensis* (Bisht and Mukai 2002; Devarumath *et al.* 2010). On the contrary, Neves *et al.* (2005) proposed *E. kigeziensis* to be autotetraploid, with *E. indica* being closely related to *E. kigeziensis* but not the direct genome donor to *E. kigeziensis*. Liu *et al.* (2011) have concluded that all three tetraploids (*E. coracana, E. africana and E. kigeziensis*) are of allotetraploid origin. They suggested independent origins of *E. kigeziensis* and *E. africana*—*E. coracana*. They are of the view that both events may have involved the diploids *E. indica* and *E. tristachya* as maternal parents, but the paternal parents remain unidentified. *Eleusine indica* and *E. tristachya* are considered to be very similar and the degree of relationship between the two remains unresolved (Hiremath and Chennaveeraiah 1982; Hiremath and Salimath 1991; Hilu and Johnson 1992; Werth *et al.* 1994; Bisht and Mukai 2001*a*, *b*; Neves *et al.* 2005; Liu *et al.* 2011).

In the present study, the cpDNA restriction site pattern variation of seven amplified chloroplast genes/intergenic spacers, the chloroplast *trn*K gene sequence and cp microsatellite polymorphism were investigated for the first time in all nine *Eleusine* species, with the objective of constructing the phylogeny of the genus *Eleusine* and identifying the maternal genome donors of the polyploid species including *E. coracana*. Both direct sequencing of the *trn*K gene and polymerase chain reaction–restriction fragment length polymorphism (PCR–RFLP) of cpDNA regions and chloroplast simple sequence repeats (cpSSRs) are considered to be very good markers for detecting cpDNA variation. The chloroplast *trn*K gene (which also contains the *mat*K gene within the *trn*K gene intron) sequence has been effectively used for the construction of grass phylogenies (Hilu and Alice 1999; Hilu *et al.* 1999). The *mat*K gene sequence is one of the seven loci widely utilized for the DNA barcoding of plants (Babbar *et al.* 2012). Specific chloroplast genes and/or intergenic spacers can be amplified (Taberlet *et al.* 1991; Demesure *et al.* 1995; Tsumura *et al.* 1995, 1996; Dhingra and Folta 2005; Heinze 2005). The amplicons can be directly sequenced or restriction endonuclease digested (PCR–RFLP or cleaved amplified polymorphic sequence), and the occurrence of microsatellites (cpSSRs) in the chloroplast genome has been widely utilized for species identification, reconstruction of phylogenetic relationships, taxonomic studies and the identification of maternal parents in polyploids (Tsumura *et al.* 1995, 1996; Weising and Gardner 1999; Ishii and McCouch 2000; Lakshmi *et al.* 2000; Parani *et al.* 2000, 2001; Komatsu *et al.* 2001; Provan *et al.* 2001; Kishimoto *et al.* 2003; Nwakanma *et al.* 2003; Zhu *et al.* 2003; Asadi Abkenar *et al.* 2004, 2008; Van Droogenbroeck *et al.* 2004; Ibrahim *et al.* 2007; Angioi *et al.* 2008; Sehgal *et al.* 2008; Jena *et al.* 2009; Liu *et al.* 2011; Poczai *et al.* 2011).

## Methods

### Plant materials

Seeds of the *Eleusine* species and one outgroup species (*Dactyloctenium aegyptium*) utilized in the present study were obtained from the United States Department of Agriculture (USDA) (Beltsville, MD, USA), the International Livestock Research Institute (ILRI) (Addis Ababa, Ethiopia), Dr Mathews M. Dida (Maseno University, Maseno, Kenya) and the National Bureau of Plant Genetic Resources (NBPGR) (New Delhi, India). The accession numbers and source of the seed material are given in Table 1. The seeds were grown under controlled conditions in the experimental field of the Department of Botany, University of Delhi.

### DNA extraction

The total genomic DNA of the *Eleusine* species and *D. aegyptium* was isolated from fresh young leaves using the cetyl trimethyl ammonium bromide method as described by Murray and Thompson (1980) with some modifications. Instead of a CsCl–ethidium bromide ultracentrifugation step, the DNA was purified by phenol–chloroform extraction.

### PCR amplification of chloroplast genes/intergenic spacers, their digestion and data analysis

Seven chloroplast genes and intergenic spacers (*trn*S–*psb*C, *psa*A, *16*S, *trn*K, *psb*D, *trn*L–*trn*F and *trn*H–*trn*K) were amplified using previously published universal forward and reverse primers (Table 2). Amplification was carried out in 100 μL reaction mixtures containing 80 ng of template DNA, 0.1 mM dNTPs (Amersham Biosciences, UK), 2 mM MgCl_2_ (Bangalore Genei, India), 1.3 μM each forward and reverse primer (Bangalore Genei), 2.5 U of *Taq* DNA polymerase (Bangalore Genei) and 10 μL of 10× assay buffer [100 mM Tris pH 9.0, 500 mM KCl, 0.1 % gelatin (Bangalore Genei)]. DNA amplification was performed in a My cycler (Bio-Rad, USA) programmed to 36 cycles each of 1 min (5 min for the first cycle) at 94 °C for template DNA denaturation, 1 min at the annealing temperature (63 °C for *psa*A, 55 °C for *trn*S–*psb*C, *trn*K and *psb*D, 60 °C for *16*S, and 50 °C for *trn*L–*trn*F and *trn*H–*trn*K), and 2 min at 72 °C for primer extension, followed by a final extension cycle of 15 min at 72 °C.

**Table 2.** List of chloroplast genes/intergenic spacers amplified in the present study. ^a^Sizes in source.

Genes/intergenic spacers	Primer pair	Size in bp^a^	Source
*trn*S–*psb*C	5′-GGTTCGAATCCCTCTCTCTC-3′ 5′-GGTCGTGACCAAGAAACCAC-3′	1600	*Oryza sativa* (Demesure *et al.* 1995)
*psa*A	5′-AAGAATGCCCATGTTGTGGC-3′ 5′-TTCGTTCGCCGGAACCAGAA-3′	2218	*Nicotiana tabacum* (Shinozaki *et al.* 1986; Tsumura *et al.* 1996)
*16*S	5′-ACGGGTGAGTAACGCGTAAG-3′ 5′-CTTCCAGTACGGCTACCTTG-3′	1375	*Nicotiana tabacum* (Shinozaki *et al.* 1986; Tsumura *et al.* 1995, 1996)
*trn*K	5′-AACCCGGAACTAGTCGGATG-3′ 5′-TCAATGGTAGAGTACTCGGC-3′	2569	*Oryza sativa* (Hiratsuka *et al.* 1989; Tsumura *et al.* 1995, 1996)
*psb*D	5′-TATGACTATAGCCCTTGGTA-3′ 5′-TAGAACCTCCTCAGGGAATA-3′	1042	*Nicotiana tabacum* (Shinozaki *et al.* 1986; Tsumura *et al.* 1995, 1996)
*trn*L–*trn*F	5′-CGAAATCGGTAGACGCTACG-3′ 5′-ATTTGAACTGGTGACACGAG-3′	995	*Nicotiana tabacum* (Taberlet *et al.* 1991)
*trn*H–*trn*K	5′-ACGGGAATTGAACCCGCGCA-3′ 5′-CCGACTAGTTCCGGGTTCGA-3′	1831	*Nicotiana tabacum* (Demesure *et al.* 1995)

Seven genes and intergenic spacers amplified from the chloroplast genome were separately restricted with 31 four-, five- and six-base cutter restriction endonucleases (AluI, AvaI, AccI, AfaI, BamHI, BalI, BglI, BglII, ClaI, DraI, EcoRV, EcoRI, HaeIII, HinfI, HindIII, HincII, MspI, KpnI, MboI, MluI, PstI, PvuII, SacI, SalI, SpeI, SphI, SmaI, SspI, TaqI, XhoI and XbaI). The 12.20 μL reaction mix contained 10 μL of the amplified gene product, 1.2 μL of enzyme buffer and 10 U of the restriction enzyme. After gentle mixing the mixture was incubated overnight at 37 °C (except for TaqI, which was incubated at 60 °C). The digestion was terminated by adding 1.5 μL of 10× loading buffer. The digested products were fractionated on 1.5 % agarose gels containing ethidium bromide (0.05 μg mL^−1^) in 0.5× TBE buffer. A DNA ladder mix was loaded alongside the digested products to serve as size markers. After agarose gel electrophoresis, the gel was photographed in ultraviolet light. Reproducibility of the patterns was tested by repeating all the reactions at least twice.

For PCR–RFLP analysis, the presence (1) or absence (0) of a restriction fragment was recorded. Total and mean character differences between pairs of species were calculated using PAUP* 4.0 (Swofford 2002). Nei and Li's coefficient of genetic distance (Nei and Li 1979) was calculated between each pair of species after the optimality criterion was set to DISTANCE. Cluster analysis was carried out using the unweighted pair-group method using arithmetic averages (UPGMA) (Sneath and Sokal 1973) and neighbour-joining (NJ) (Saitou and Nei 1987) methods. Bootstrap values were calculated from 100 replicates using the BOOTSTRAP command in PAUP.

### PCR amplification, cloning and sequencing of the *trn*K gene, and data analysis

The *trn*K gene was amplified from the nine *Eleusine* species and one outgroup as described above. The amplification products were separated on a 1 % agarose gel, excised from the gel and purified using a QIA quick gel extraction kit (Qiagen, Germany). The purified amplification products were cloned and sequenced with an ABI PRISM 377 automated DNA sequencer (Applied Biosystems, Foster City, CA, USA). BLAST similarity searches were performed using the National Centre for Biotechnology Information (NCBI) BLASTN algorithm to confirm the identity of the *trn*K sequences. The nucleotide sequences have been submitted to GenBank with accession numbers KF357736–KF357745. The nucleotide sequence of the partial *trn*K gene from *Acrachne racemosa* available in GenBank (accession number JN681616.1) was also utilized for phylogenetic analysis. The consensus sequence for the *trn*K gene from the nine *Eleusine* taxa and one outgroup (*D. aegyptium*) was generated using the software CLC Sequence viewer 6.1.

The sequences were aligned using Clustal-X, version 1.8 (Thompson *et al.* 1997). Phylogenetic analyses were carried out with the software MEGA 5 (Tamura *et al.* 2011). Pairwise sequence divergence rates between species were calculated using the maximum composite likelihood method. Phylogeny construction was carried out using NJ, minimum evolution (ME), maximum likelihood (ML) and maximum parsimony (MP) methods. Neighbour-joining and ME trees were obtained using the maximum composite likelihood criterion while ML and MP trees were constructed using the nearest-neighbour-interchange (NNI) and tree-bisection-reconnection algorithms, respectively. In all the analyses, all positions containing gaps and missing data were eliminated from the dataset (complete deletion option). Support values of the internal branches of NJ, ME, ML and MP trees were evaluated by bootstrap (500 replicates).

### PCR amplification of chloroplast microsatellites, polyacrylamide gel electrophoresis and sequencing

A total of eight primer pairs (Table 3) were used for amplification of the chloroplast microsatellites. Amplification was carried out in 25 mL reaction mixtures containing 2.5 mL of 10× reaction buffer, 1.5 mM MgCl_2_, 200 mM dNTPs, 200 nM each primer, 0.5 U of *Taq* DNA polymerase (Bangalore Genei) and 25 ng of template DNA. DNA amplification was performed in a MyCycler™ (Bio-Rad) programmed to initial denaturation at 94 °C for 5 min followed by 35 cycles each of 1 min at 94 °C, 1 min at 55 °C (65 °C for ccmp2 and 53 °C for ccmp9) and 1 min at 72 °C, followed by a final extension cycle of 5 min at 72 °C.

**Table 3.** List of chloroplast microsatellites amplified in the present study.

Locus	Location	Repeat	Primer pair	Size in *Eleusine* (bp)	Size in source (bp)	Source
ccmp2	5′ to *trn*S	(A)_11_	5′-GATCCCGGACGTAATCCTG-3′ 5′-ATCGTACCGAGGGTTCGAAT-3′	197, 200	189	*Nicotiana tabacum* (Weising and Gardner 1999)
ccmp5	3′ to *rps2*	(C)_7_(T)_10_ (T)_5_C(A)_11_	5′-TGTTCCAATATCTTCTTGTCATTT-3′ 5′-AGGTTCCATCGGAACAATTAT-3′	145, 146	103	*Nicotiana tabacum* (Weising and Gardner 1999)
ccmp6	ORF77–ORF82 intergenic	(T)_5_C(T)_17_	5′-CGATGCATATGTAGAAAGCC-3′ 5′-CATTACGTGCGACTATCTCC-3′	96	98	*Nicotiana tabacum* (Weising and Gardner 1999)
RCt3	Intergenic region	(A)_10_	5′-TAGGCATAATTCCCAACCCA-3′ 5′-CTTATCCATTTGGAGCATAGGG-3′	113	129	*Oryza sativa* cv Nipponbare (Ishii and McCouch 2000)
RCt4	Coding region (*psb*G)	(T)_12_	5′-ACGGAATTGGAACTTCTTTGG-3′ 5′-AAAAGGAGCCTTGGAATGGT-3′	131	128	*Oryza sativa* cv Nipponbare (Ishii and McCouch 2000)
RCt5	Intergenic region	(T)_10_	5′-ATTTGGAATTTGGACATTTTCG-3′ 5′-ACTGATTCGTAGGCGTGGAC-3′	151	143	*Oryza sativa* cv Nipponbare (Ishii and McCouch 2000)
RCt7	Coding region (*inf*A)	(T)_10_	5′-GTGTCATTCTCTAGGCGAAC-3′ 5′-AAATATGACAGAAAAGAAAAATAGG-3′	126	126	*Oryza sativa* cv Nipponbare (Ishii and McCouch 2000)
RCt8	Intron (*rpl*16)	(T)_17_	5′-ATAGTCAAGAAAGAGGATCTAGAAT-3′ 5′-ACCGCGATTCAATAAGAGTA-3′	125	131	*Oryza sativa* cv Nipponbare (Ishii and McCouch 2000)

An equal volume (10 mL) of formamide dye (98 % formamide, 10 mM EDTA, 0.026 g of bromophenol blue, 0.026 g of xylene cyanol) was added to each amplified product. The samples were heated for 5 min at 94 °C and immediately placed on ice. A total of 2.5 mL of each sample was loaded on a 6 % polyacrylamide gel (19 : 1 acrylamide : bisacrylamide, 7.5 M urea and 1× TBE buffer), and electrophoresis was conducted at 55 W and 55 °C for ∼2 h.

For silver staining, the gel was fixed in 10 % (v:v) acetic acid for 30 min. It was subsequently rinsed three times in de-ionized water (2 min per rinse). The gel was then kept for staining for 30 min in a 2 L solution containing 2 g of silver nitrate and 3 mL of 37 % formaldehyde (Promega, USA). The stained plate was rinsed with de-ionized water for 20 s and developed in a prechilled (10 °C) developer (2 L) solution containing 60 g of sodium carbonate, 3 mL of 37 % formaldehyde and 400 µL of sodium thiosulfate (10 mg mL^−1^). When bands became visible, the gel was immediately transferred to 10 % acetic acid solution to stop further reaction. The gel was finally rinsed with distilled water and air dried.

The PCR products of eight primer–template combinations were characterized by direct sequencing. A PCR was performed in 100 µL volumes as described above. An aliquot of the PCR product was checked by agarose gel electrophoresis, and the remainder was purified through a QIA quick PCR clean up kit (Qiagen). Nucleotide sequencing was performed using an ABI PRISM 377 automated DNA sequencer (Applied Biosystems). The nucleotide sequences have been submitted to GenBank with accession numbers KF357730–KF357735.

## Results

### Chloroplast PCR–RFLP

**Figure 1.**
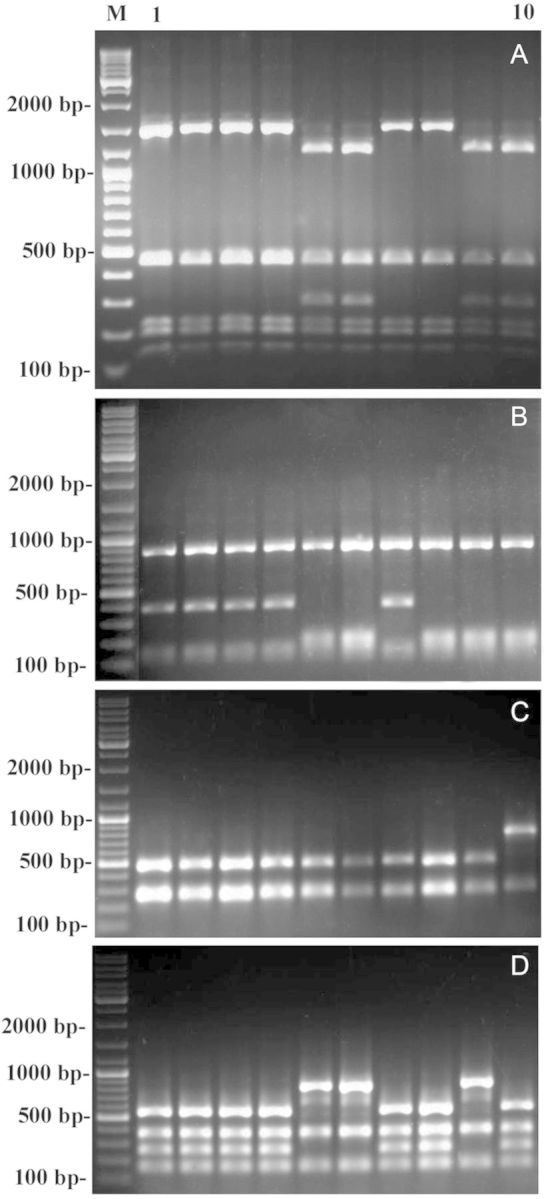
Restriction fragment size patterns of the amplified *trn*K gene with AfaI (A), the amplified *trn*S*–psb*C intergenic spacer with AfaI (B), the amplified *trn*L*–trn*F gene with MboI (C) and the amplified *16*S gene with MboI (D) in *E. coracana* (lane 1), *E. africana* (2), *E. tristachya* (3), *E. indica* (4), *E. floccifolia* (5), *E. intermedia* (6), *E. kigeziensis* (7), *E. multiflora* (8), *E. jaegeri* (9) and *D. aegyptium* (10). Marker DNA (M). The size of the fragments in base pairs is indicated on the left.

Robust amplification products were obtained for all seven genes/intergenic spacers (*trn*S–*psb*C, *psa*A, *16*S, *trn*K, *psb*D, *trn*L–*trn*F and *trn*H–*trn*K) from the nine *Eleusine* species and an outgroup species, *D. aegyptium*. For each gene/intergenic spacer, the amplified products appeared to be monomorphic on a 1.5 % agarose gel across the species. The approximate size of the PCR product for *trn*S–*psb*C, *psa*A, *16*S, *trn*K, *psb*D, *trn*L–*trn*F and *trn*H–*trn*K was 1620, 2380, 1480, 2510, 1090, 1090 and 2270 bp, respectively. Aliquots of PCR products were digested separately with 31 four-, five- and six-cutter restriction endonucleases. Of the 217 amplification product–enzyme combinations, 137 did not show internal restriction sites in any of the 10 species investigated. In the remaining 80 amplification product–enzyme combinations, 1–5 restriction sites within the amplification product were obtained. Of these, 57 combinations revealed no polymorphism among the 10 species. The remaining 23 (*trn*K/AfaI, *trn*K/MspI, *trn*K/DraI, *trn*K/SpeI, *trn*K/SspI, *trn*K/KpnI, *trn*K/TaqI, *trn*K/BamHI, *trn*L–*trn*F/AluI, *trn*L–*trn*F/BglII, *trn*L–*trn*F/DraI, *trn*L–*trn*F/MboI, *psa*A/AluI, *psa*A/TaqI, *psa*A/MspI, *psb*D/HaeIII, *psb*D/MboI, *psb*D/TaqI, *trn*S–*psb*C/AfaI, *trn*S–*psb*C/AvaI, *trn*S–*psb*C/HaeIII, *16*S/MboI, *trn*H–*trn*K/AluI) combinations were phylogenetically very informative (Table 4; Fig. 1). A total of 282 bands were scored for cluster analyses. No variation was observed across different accessions of the same taxon. The polymorphism in all the profiles was the result of site mutations.

**Table 4.** Grouping of *Eleusine* and outgroup species based on restriction fragment patterns of seven cpDNA amplified genes/intergenic spacers.

	Species	*trn*S–*psb*C			16S	*psb*D			*psa*A			*trn*K								*trn*H–*trn*K	*trn*L–*trn*F			
		AfaI	AvaI	HaeIII	MboI	HaeIII	MboI	TaqI	AluI	MspI	TaqI	AfaI	DraI	MspI	SpeI	SspI	KpnI	TaqI	BamHI	AluI	AluI	BglII	DraI	MboI
	*E. coracana*	A1	B1	C1	D1	E1	F1	G1	H1	I1	J1	K1	L1	M1	N1	O1	P1	Q1	R1	S1	T1	U1	V1	X1
	*E. africana*	A1	B1	C1	D1	E1	F1	G1	H1	I1	J1	K1	L1	M1	N1	O1	P1	Q1	R1	S1	T1	U1	V1	X1
	*E. tristachya*	A1	B1	C1	D1	E1	F1	G1	H1	I1	J1	K1	L1	M1	N1	O1	P1	Q1	R1	S1	T1	U1	V1	X1
	*E. indica*	A1	B1	C1	D1	E1	F1	G1	H1	I1	J1	K1	L1	M1	N1	O1	P1	Q1	R1	S1	T1	U1	V1	X1
	*E. floccifolia*	A2	B1	C1	D2	E1	F2	G1	H2	I1	J1	K2	L1	M1	N1	O2	P1	Q1	R1	S1	T1	U1	V1	X1
	*E. intermedia*	A2	B1	C1	D2	E1	F2	G1	H2	I1	J1	K2	L1	M1	N1	O2	P1	Q1	R1	S1	T1	U1	V1	X1
	*E. kigeziensis*	A1	B1	C1	D1	E1	F1	G1	H1	I1	J1	K1	L1	M1	N1	O1	P1	Q1	R1	S1	T1	U1	V1	X1
	*E. jaegeri*	A2	B1	C1	D2	E1	F2	G1	H2	I2	J1	K2	L1	M1	N1	O2	P1	Q1	R1	S1	T1	U1	V1	X1
	*E. multiflora*	A2	B1	C1	D1	E2	F2	G1	H1	I1	J2	K1	L2	M2	N2	O2	P1	Q1	R1	S1	T1	U1	V1	X1
	*D. aegyptium*	A2	B2	C2	D1	E3	F2	G2	H1	I1	J1	K2	L1	M3	N1	O1	P2	Q2	R2	S2	T2	U2	V2	X2
Combinations		I	VIII	VIII	III	IV	I	VIII	III	V	VI	VII	VI	IV	VI	II	VIII	VIII	VIII	VIII	VIII	VIII	VIII	VIII

**Figure 2.**
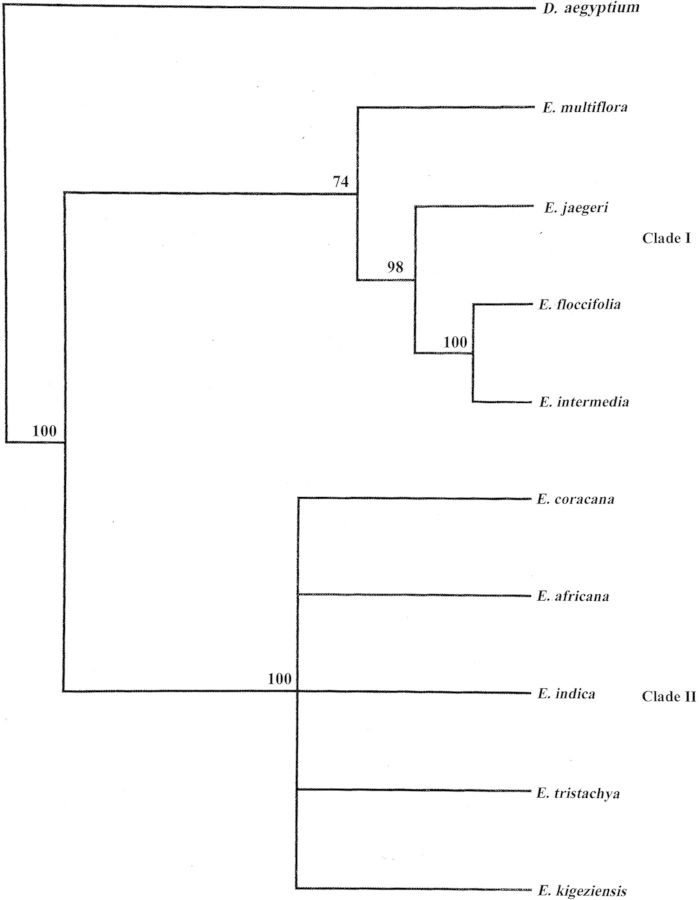
Unweighted pair-group method using arithmetic averages dendrogram based on the restriction fragment data of amplified chloroplast gene/intergenic spacers. Numbers at the nodes represent bootstrap probability values out of 100 replicates.

Genetic distance (Nei and Li's) among the nine *Eleusine* species and one outgroup ranged from 0.0000 to 0.01857 (Table 5). Based on the restriction site data of the amplified gene/intergenic products, UPGMA and NJ dendrograms were generated. Both showed similar topologies. They resolved into two major clades. Clade I, supported by 100 % bootstrap support, contained *E. kigeziensis, E. indica*, *E. tristachya, E. coracana* and *E. africana*. Clade II was comprised of *E. jaegeri*, *E. floccifolia*, *E. intermedia* and *E. multiflora. Eleusine floccifolia* and *E. intermedia* were more closely related to each other (supported by a bootstrap value of 100 %) than either of the species was to *E. jaegeri. Dactyloctenium aegyptium* was the most diverged species among the analysed species (Fig. 2).

**Table 5.** Genetic distance (Nei and Li's coefficient) matrix generated from cpDNA PCR–RFLP data of nine *Eleusine* species and an outgroup, *D. aegyptium*.

	1	2	3	4	5	6	7	8	9	10
1	*E. coracana*									
2	*E. africana*	0.00000								
3	*E. tristachya*	0.00000	0.00000							
4	*E. indica*	0.00000	0.00000	0.00000						
5	*E. floccifolia*	0.00479	0.00479	0.00479	0.00479					
6	*E. intermedia*	0.00479	0.00479	0.00479	0.00479	0.00000				
7	*E. kigeziensis*	0.00000	0.00000	0.00000	0.00000	0.00479	0.00479			
8	*E. multiflora*	0.00650	0.00650	0.00650	0.00650	0.00790	0.00790	0.00650		
9	*E. jaegeri*	0.00655	0.00655	0.00655	0.00655	0.00170	0.00170	0.00655	0.00827	
10	*D. aegyptium*	0.01443	0.01443	0.01443	0.01443	0.01815	0.01815	0.01443	0.01692	0.01857

### *trn*K sequence data

The *trn*K gene codes for tRNA-Lys^UUU^. In rice, the chloroplast *trn*K gene is 2576 bp long, of which the exon is only 72 bp long. The exon is divided into two parts by a long intron of ∼2500 bp. The 5′ exon consists of 37 bp and the 3′ exon consists of 35 bp. The *mat*K gene, which codes for maturaseK, is 1536 bp in length and is located within the chloroplast *trn*K intron. In the present study, the *trn*K gene was sequenced from the nine *Eleusine* species and *D. aegyptium*. The aligned *trn*K gene sequence of *Eleusine* corresponded to position 45 at the 5′ end to position 2531 at the 3′ end of rice. The length of the *trn*K gene in the nine *Eleusine* species ranged between 2463 (*E. jaegeri*) and 2467 bp (*E. tristachya* and *E. floccifolia*). The length of *trn*K in *A. racemosa* and *D. aegyptium* was 2466 and 2472 bp, respectively **[see****Supporting Information****]**. The GC content of the *trn*K gene sequence varied from 32.3 to 33 % with an average of 32.6 %. The nucleotide frequencies were 0.162 for G, 0.164 for C, 0.324 for A and 0.350 for T. The dataset including alignment gaps and missing data comprised 2486 nucleotide positions, out of which 2254 were conserved, 214 were variable and 57 were parsimony informative sites.

**Figure 3.**
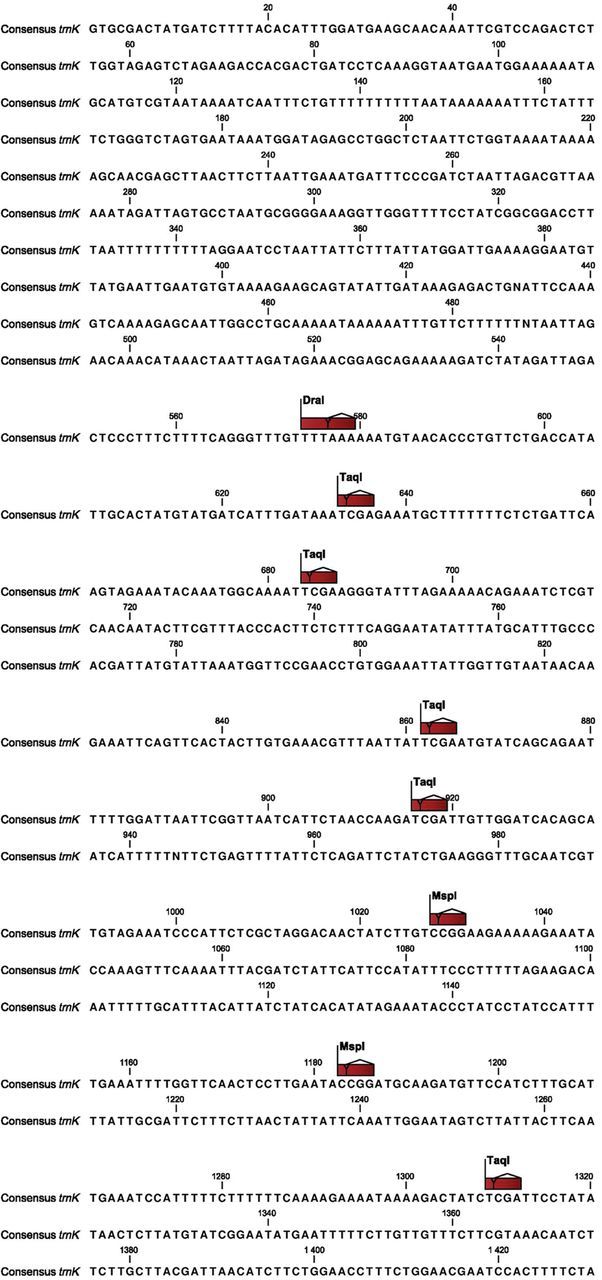

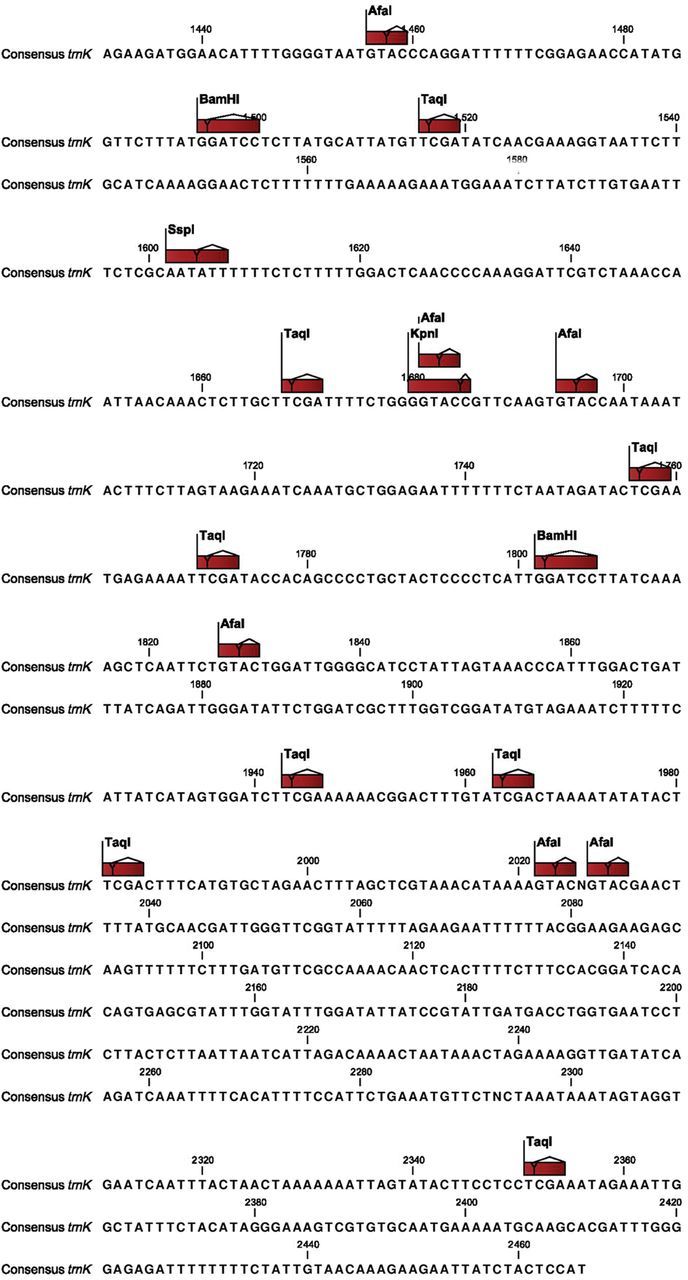
Consensus sequence of the *trn*K gene for nine *Eleusine* species and one outgroup (*D. aegyptium*) showing major restriction sites.

The *trn*K sequence data were correlated with the polymorphic PCR–RFLP profiles of the *trn*K gene. Eight amplicon–enzyme combinations for the *trn*K gene showed polymorphic profiles which revealed that polymorphism in all the profiles was the result of site mutations. The entire *trn*K gene sequence from the taxa analysed showed 214 single-nucleotide polymorphisms (SNPs). A total of 10 SNPs in the restriction sites were responsible for the eight polymorphic profiles. The polymorphic profiles produced were due to gain or loss of the restriction sites caused by SNPs. The consensus *trn*K gene sequence was generated from the taxa analysed and the locations of the polymorphic sites were marked on it (Fig. 3).

**Figure 4.**
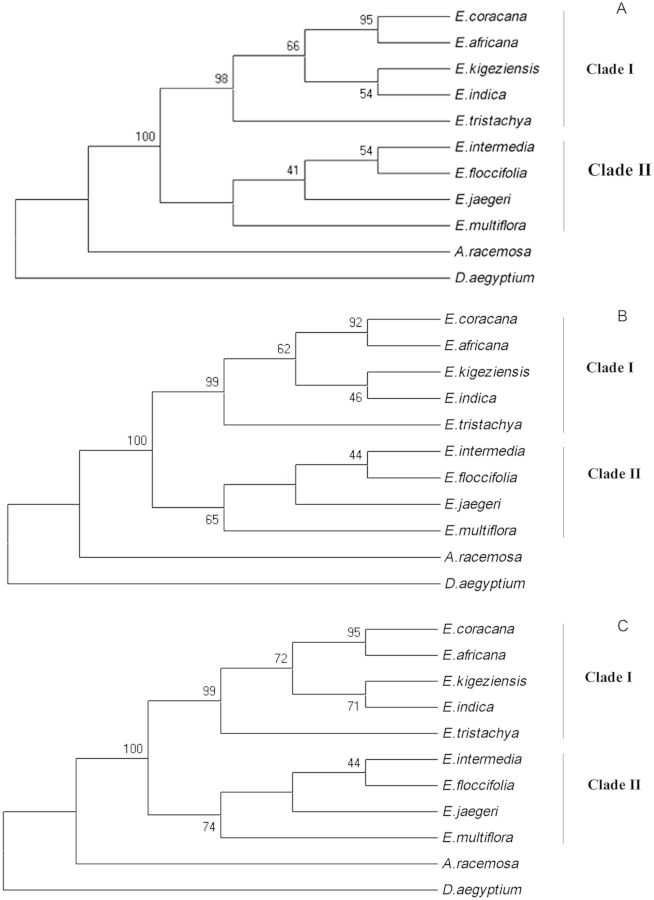
Dendrograms based on the sequence data of the *trn*K gene of *Eleusine* and outgroup taxa. (A) Neighbour-joining bootstrap consensus tree, (B) ML bootstrap consensus tree and (C) MP consensus tree. Numbers at the nodes represent bootstrap probability values out of 500 replicates.

Pairwise sequence divergence ranged from 0.003 to 0.058 (Table 6). Dendrograms were generated using four different methods (NJ, ML, ME and MP). The phylogeny reconstruction through the NJ method resulted in an optimal tree with a sum of branch length of 0.095. In the NJ bootstrap consensus tree, all the *Eleusine* species were grouped into two distinct clades with 100 % bootstrap support. Clade I consisted of *E. coracana*, *E. africana*, *E. indica*, *E. kigeziensis* and *E. tristachya. Eleusine coracana* and *E. africana* grouped together in one subclade with a bootstrap support of 95 %, and *E. indica* and *E. kigeziensis* grouped together in another subclade with a bootstrap support of 54 % within Clade I. *Eleusine tristachya* did not group within any of the two subclades and thus was the most diverged among the five species of Clade I with a bootstrap support of 98 %. Clade II consisted of *E. intermedia*, *E. floccifolia*, *E. jaegeri* and *E. multiflora. Eleusine intermedia* and *E. floccifolia* were more closely related to each other than to *E. jaegeri* with a bootstrap support of 54 %. *Eleusine multiflora* represented the most diverged species within Clade II. *Acrachne racemosa* and *D. aegyptium* were the most diverged species among all the species (Fig. 4A).

**Table 6.** Genetic divergence (maximum composite likelihood method) from *trn*K sequence data for nine *Eleusine* species and two outgroups, *A. racemosa* and *D. aegyptium.*

1	2	3	4	5	6	7	8	9	10	11
*E. floccifolia*										
*E. intermedia*	0.008									
*E. jaegeri*	0.026	0.025								
*E. multiflora*	0.010	0.009	0.027							
*E. coracana*	0.012	0.012	0.026	0.012						
*E. africana*	0.013	0.013	0.028	0.013	0.004					
*E. kigeziensis*	0.014	0.014	0.030	0.014	0.007	0.008				
*E. indica*	0.011	0.010	0.026	0.010	0.003	0.004	0.005			
*E. tristachya*	0.011	0.011	0.026	0.011	0.005	0.006	0.007	0.003		
*A. racemosa*	0.029	0.028	0.046	0.029	0.030	0.030	0.030	0.027	0.027	
*D. aegyptium*	0.039	0.038	0.058	0.041	0.040	0.041	0.043	0.039	0.040	0.030

The robustness of the present data lies in the fact that the dendrograms based on ME, ML and MP analysis showed almost the same topology as that of the NJ tree (Fig. 4). Maximum parsimony analysis resulted in most parsimonious trees (Fig. 4C) with a tree length of 232. The bootstrap consensus tree had a consistency index of 0.943, a retention index of 0.859 and a rescaled consistency index of 0.811.

### Chloroplast microsatellite polymorphism and sequence data

**Figure 5.**
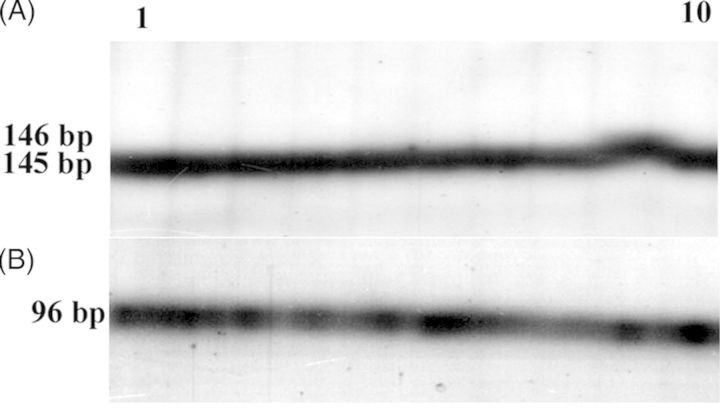
Silver-stained polyacrylamide gel electrophoresis patterns generated by ccmp5 (A) and ccmp6 (B) in *E. coracana* (1), *E. africana* (2), *E. tristachya* (3), *E. indica* (4), *E. floccifolia* (5), *E. intermedia* (6), *E. kigeziensis* (7), *E. multiflora* (8), *E. jaegeri* (9) and *D. aegyptium* (10). The size of the fragments in base pairs is indicated on the left.

Eight consensus primer pairs were used to amplify cpDNA microsatellites from the nine species (Table 3). The primer pairs were able to amplify a product in all 10 species (Fig. 5). A major band was produced for each primer–template combination. The typical stuttering phenomenon which usually occurs upon amplification of mononucleotide- and dinucleotide-type microsatellites was observed. Size variation for the amplified products was only observed for two cpSSR loci. Two size variants were detected for ccmp2 and ccmp5. For ccmp2, all but one (*E. tristachya*) species showed a 197-bp allele. In *E. tristachya*, it was 200 bp. In the case of ccmp5, *E. jaegeri* showed a band of 146 bp, while each of the remaining nine species had a 145-bp allele (Fig. 5).

To test the presence of a repeat motif in *Eleusine*, nucleotide sequencing was carried out for amplified products from *E. coracana* with all eight cpSSR primer sets. RCt5 and RCt8 could not be successfully sequenced. ccmp2, ccmp5, ccmp6, RCt3, RCt4 and RCt7 showed sequence sizes of 197, 145, 96, 113, 131 and 126 bp, respectively (Tables 3 and 7). Repeat motifs were identified in all six amplification products at the expected positions but the degree of conservation was variable (Table 7). Among the repeat motifs, RCt4 and RCt7 were highly conserved, ccmp5 and ccmp6 were moderately conserved, and ccmp2 and RCt3 were least conserved. In the case of ccmp2 and RCt3, the number of mononucleotide repeats was reduced.

**Table 7.** Nucleotide sequences of cpDNA of *E. coracana* amplified using six cpSSR primer pairs. The location of repeats is underlined.

ccmp2 (complete sequence)
5′-GATCCCGGACGTAATCCTGGACGTATCCTGGACGTGAGGAGTAAAAATCCAAAATTTTTGGGAATTTTTTCTTACAAATTGAATTTATTTCGTACATTTATCTATGAA AAAATCCGGGGGTTAGAATTCCTTACAATTCGAAAGTCCCAAACGATCCGAGGGGGCGGAAAGAGAGGGATTCGAACCCTCGGTACGAT-3′
ccmp5 (complete sequence)
5′-TGTTCCAATATCTTCTTGTCATTTTTTCCACACTTCCTTTTTTTTTTCTTTTTTTCGTCTTACCATTATGGAATTTTTTTCTTTTTGAAGATTAAGAAAGAGCCAAATTAT CTTGAAATAAATAATAATTGTTCCGATGGAACCT-3′
ccmp6 (complete sequence)
5′-CGATGGATATGTAGAAAGCCCTTTTTCTAGTATTTACTAGAAAATTCATCTTTTTTTCTTCTTCTCTTTCTATAGTGGAGATAGTCGCACGTAATG-3′
RCt3 (partial sequence)
5′-TTCTATCACAaaAATAACATAAAAACTTATAAATTGCTCCCTATGCTCCAAATGGATAAG-3′
RCt4 (complete sequence)
5′-ACGGAATTGGAACTTCTTTGGTCCAGTAACGGGAAATCCATCCAAACTTCCTGGCCGTTTTCCATGGAATCTTTTCCTTCTTTTTTTTTTTTGGCGGAATATCCGGTA AAAACCATTCCAAGGCTCCTTTT-3′
RCt7 (complete sequence)
5′-GTGTCATTCTCTAAGCGAACTCGGAACATTCCGTTGGGTAGGGCTTCCGTAACTAAACCTTCGAAAGTTACTTTTGCTTCTCTCGGGTTTTTTTTTTCTCTCCTATTTT TTTTTTCTGTCATGTTT-3′

## Discussion

The restriction site variation identified in the amplified chloroplast gene/intergenic spacers has provided new insights into the origin and evolution of the three polyploid species, and the genetic relationships between the cultivated and wild *Eleusine* species. Species-specific markers were also identified. None of the amplicon–enzyme combinations could discriminate between *E. coracana* (AABB, 2*n* = 4*x* = 36), *E. africana* (AABB, 2*n* = 4*x* = 36), *E. tristachya* (AA, 2*n* = 2*x* = 18), *E. indica* (AA, 2*n* = 2*x* = 18) and *E. kigeziensis* (AADD, 2*n* = 4*x* = 38) on the one hand, and between *E. floccifolia* (BB, 2*n* = 2*x* = 18) and *E. intermedia* (AB, 2*n* = 2*x* = 18) on the other hand. Although the maximum number of polymorphic and phylogenetically informative markers was obtained in eight *trn*K amplicon–enzyme combinations, the amount of variation that can be tapped with the PCR–RFLP method was lower than the variation tapped with sequencing of the *trn*K gene. A total of 214 SNPs were found in the *trn*K gene sequence, of which only 10 were causative to 8 polymorphic profiles. This clearly demonstrates that sequencing of the *trn*K gene is more informative than the PCR–RFLP method. Moreover, the nucleotide sequence of the chloroplast *trn*K gene fine-tuned the species relationships. The nine species of *Eleusine* were grouped into two clades, in both PCR–RFLP and *trn*K gene sequence data analyses. The *trn*K sequence data clearly showed that the three tetraploids were closer to *E. indica* than to *E. tristachya*, as concluded from PCR–RFLP data.

The present observations based on restriction site variation and the *trn*K gene sequence of cpDNA are congruent with the conclusions reached by various authors based on the multiple marker nuclear DNA assay, chloroplast markers and chromosome research. The strong affinities between *E. coracana*, *E. africana* and *E. indica* have been highlighted in earlier studies (Hilu and Johnson 1992, 1997; Hilu 1995; Bisht and Mukai 2000, 2001*a*, *b*; Neves *et al.* 2005; Dida *et al.* 2007, 2008; Liu *et al.* 2011) as well. A number of studies based on chromosome research (Hiremath and Chennaveeraiah 1982; Hiremath and Salimath 1991), 2C DNA content (Hiremath and Salimath 1991), crossability data (Salimath *et al.* 1995*b*) and ribosomal DNA polymorphism (Hilu and Johnson 1992; Werth *et al.* 1994; Bisht and Mukai 2000), ITS sequence data (Neves *et al.* 2005) and cpDNA (Hilu and Johnson 1997; Neves *et al.* 2005; Liu *et al.* 2011) also support the close affinity between *E. indica* and *E. tristachya*. The allotetraploid *E. kigeziensis* exhibits the same maternal lineage as that of the two other allotetraploid species, *E. coracana* and *E. africana*. The five species share some common morphological features, such as 1–3 nerved lower glumes with a winged keel, 3–7 nerved upper glumes with a more or less winged keel, the presence of 1–3 subsidiary nerves adjacent to the central nerve of the lemma and the generally winged keel of the palea (Phillips 1972).

The present data unambiguously support the view of Chennaveeraiah and Hiremath (1974), Hilu (1988), Hiremath and Salimath (1992), Hilu and Johnson (1997), Bisht and Mukai (2000, 2001*a*, *b*) and Neves *et al.* (2005) that *E. africana* is the wild progenitor of the cultivated species, *E. coracana*, and that *E. indica* is one of the diploid progenitors of the two polyploid species (Liu *et al.* 2011). The present results further indicate that *E. indica* with the AA genome might be the maternal parent for all three tetraploid species, viz. *E. coracana*, *E. africana* and *E. kigeziensis.* Morphologically, *E. kigeziensis* resembles *E. indica*. It is considered to be a hybrid between *E. indica* and one of the perennial species (Phillips 1972). Salimath (1990) and Bisht and Mukai (2002) have also proposed *E. indica* as one of the genome donors of *E. kigeziensis*.

The close affinity in the cpDNA PCR–RFLP profiles and *trn*K sequences between *E. floccifolia* and *E. intermedia* is also supported by the occurrence of the same chromosome number (2*n* = 18), 2C DNA values (Hiremath and Chennaveeraiah 1982; Hiremath and Salimath 1991) and ITS sequence data (Neves *et al.* 2005). Close association between *E. jaegeri*, *E. floccifolia* and *E. intermedia* suggested in earlier studies (Hiremath and Salimath 1991; Hilu and Johnson 1992; Liu *et al.* 2011) is further supported by our data. *Eleusine jaegeri* in all combinations grouped together with *E. floccifolia* and *E. intermedia* except in the *psa*A–MspI combination, where it shows a unique profile *vis-à-vis* the remaining species. In the case of *trn*K sequence analysis also these three species are found to be closely associated. *Eleusine multiflora* also behaves in the same fashion. The species exhibits unique profiles *vis-à-vis* the remaining species in *trn*K–DraI, *trn*K–MspI, *trn*K–SpeI, *psa*A–TaqI and *psb*D–HaeIII combinations, with the result that it represents the most diverged taxon among all the *Eleusine* species. These amplicon–enzyme combinations can therefore be used as species-specific markers for *E. jaegeri* and *E. multiflora*. The position of *E. multiflora* within the genus *Eleusine* is questionable mainly on the basis of its unusual inflorescence morphology [considered to be intermediate between *Eleusine* and *Acrachne* (Phillips 1972; Clayton and Renvoize 1986)], distinct chemical composition (Hilu *et al.* 1978), molecular data (Hilu and Johnson 1992; Hilu 1995), chromosome number (2*n* = 16), 2C DNA value and other cytogenetic features (Mysore and Baird 1997; Bisht and Mukai 2000) including the present results. *Dactyloctenium aegyptium* in restriction site analysis, and *A. racemosa* and *D. aegyptium* in *trn*K sequence analysis indicate that they are the most diverged species *vis-à-vis* *Eleusine* species analysed and thus represented as outgroups.

Chloroplast microsatellites are known to have potential for phylogenetic studies (Powell *et al.* 1995*a*, *b*, 1996; Provan *et al.* 1997, 2001; Ishii and McCouch 2000; Angioi *et al.* 2008). The present cpSSR data on *Eleusine* species, however, were not helpful as very little polymorphism was obtained for various amplified microsatellites. Only two cpSSR loci (ccmp2 and ccmp5) were found to be polymorphic, displaying a total of four alleles. In the case of ccmp2, the variant allele was found in *E. tristachya*, while for ccmp5 the variant allele was found in *E. jaegeri*. Overall, the sequences seem to be highly conserved. This is in strong contrast to the observations made in the other genera such as *Glycine*, *Oryza* and *Hordeum*, where different species within the genus and even within different subspecies displayed characteristic haplotypes (Powell *et al.* 1995*b*, 1996; Provan *et al.* 1997, 1999). The reason for not obtaining polymorphism in the present study could be attributed to the fact that it is very difficult to design universal primers for microsatellites that show widespread polymorphism at the inter-specific level and at the same time are able to amplify across a broad range of plant genera. Although there are primers available that amplify across wide-ranging taxa, these rarely show widespread inter-specific polymorphism. This could be due to the contrasting requirements in a short stretch of DNA of extreme stability and sequence conservation for priming sites and consistently high levels of polymorphism in the intervening region (Provan *et al.* 2001). Second, size homoplasy may not necessarily mean identical intervening microsatellite regions; it may also be due to variation in flanking regions. It is also possible that back mutations have taken place, which might result in identical size but not identical sequence (Bryan *et al.* 1999).

## Conclusions

Our results based on RFLP of the seven amplified chloroplast genes/intergenic spacers, and the *trn*K gene sequence in the nine diploid and allotetraploid *Eleusine* species and two outgroup species resulted in well-resolved phylogenetic trees. The maternal genome donor (*E. indica*, 2*n* = 2*x* = 18) of the allotetraploid (2*n* = 4*x* = 36, 2*n* = 2*x* = 38) *Eleusine* species, and the phylogenetic relationships between cultivated *E. coracana* (2*n* = 4*x* = 36) and wild species could be successfully resolved. The species-specific markers were also identified. The two diploid species *E. indica* and *E. tristachya* could not be resolved separately by PCR–RFLP of seven chloroplast genic/intergenic spacers, as not a single site change could be scored. However, the *trn*K gene sequence clearly demonstrated that *E. indica* is more closely related to all three allotetraploids as compared with *E. tristachya*. Therefore, *E. indica* is most likely the maternal parent to all three allotetraploids. *Eleusine multiflora* (2*n* = 2*x* = 16) was found to be the most diverged among all the species. The explicit identification of the maternal parent and that of the immediate wild progenitor of finger millet will be immensely useful for future genetic improvement and biotechnological programmes of the crop species.

## Sources of Funding

This work was supported by the Council of Scientific and Industrial Research (CSIR), Government of India and the National Academy of Sciences, India (NASI).

## Contributions by the Authors

R.A. was involved in planning and performing all the experiments, data analyses and manuscript writing. N.A. was involved in the data analysis. R.T. was involved in relevant research discussions. S.N.R. was involved in the planning and supervision of all the experimental work and in writing the manuscript. All authors have seen and agreed to the submitted manuscript.

## Conflicts of Interest Statement

None declared.

## Accession Numbers

The nucleotide sequences of the *trn*K gene from the nine *Eleusine* species and one outgroup *D. aegyptium* have been submitted to GenBank with accession numbers KF357736–KF357745. The nucleotide sequences of six cpSSRs isolated from *E. coracana* have been submitted to GenBank with accession numbers KF357730–KF357735.

## Supporting Information

The following additional information is available in the online version of this article –

**File 1.** Sequence data matrix of the aligned partial *trn*K gene region of cpDNA of nine *Eleusine* species and two outgroups, *A. racemosa* and *D. aegyptium*. Nucleotide sequences are displayed 5′–3′. Dots indicate the same nucleotide as in *E. coracana*; dashes indicate gaps.

Additional Information

## References

[PLT056C1] Angioi SA, Desiderio F, Rau D, Bitocchi E, Attene G, Papa R (2008). Development and use of chloroplast microsatellites in *Phaseolus* spp. and other legumes. Plant Biology.

[PLT056C2] Asadi Abkenar A, Isshiki S, Tashiro Y (2004). Phylogenetic relationships in the ‘true citrus fruit trees’ revealed by PCR–RFLP analysis of cpDNA. Scientia Horticulturae.

[PLT056C3] Asadi Abkenar A, Isshiki S, Matsumoto R, Tashiro Y (2008). Comparative analysis organelle DNAs in acid citrus grown in Japan using PCR–RFLP method. Genetic Resources and Crop Evolution.

[PLT056C4] Babbar SB, Raghuvanshi S, Singh HK, Parveen I, Malik S (2012). An overview of the DNA barcoding of plants. Phytomorphology.

[PLT056C5] Barbeau WE, Hilu KW (1993). Protein, calcium, iron and amino acid content of selected wild and domesticated cultivars of finger millet. Plant Foods for Human Nutrition.

[PLT056C6] Bisht MS, Mukai Y (2000). Mapping of rDNA on the chromosomes of *Eleusine* species by fluorescence *in situ* hybridization. Genes and Genetic Systems.

[PLT056C7] Bisht MS, Mukai Y (2001a). Genomic in situ hybridization identifies genome donor of finger millet (*Eleusine coracana*). Theoretical and Applied Genetics.

[PLT056C8] Bisht MS, Mukai Y (2001b). Identification of genome donors to the wild species of finger millet, *Eleusine africana* by genomic *in situ* hybridization. Breeding Science.

[PLT056C9] Bisht MS, Mukai Y (2002). Genome organization and polyploid evolution in the genus *Eleusine* (Poaceae). Plant Systematics and Evolution.

[PLT056C10] Bryan GJ, McNicoll JM, Ramsay G, Meyer RC, DeJong WQS (1999). Polymorphic simple sequence repeat markers in chloroplast genomes of solanaceous plants. Theoretical and Applied Genetics.

[PLT056C11] Chandrashekar A (2010). Finger millet *Eleusine coracana*. Advances in Food & Nutrition Research.

[PLT056C12] Chennaveeraiah MS, Hiremath SC (1974). Genome analysis of *Eleusine coracana* (L.) Gaertn. Euphytica.

[PLT056C13] Clayton WD, Renvoize SA (1986). Genera Graminum: grasses of the world.

[PLT056C14] Demesure B, Sodzi N, Petit RJ (1995). A set of universal primers for amplification of polymorphic non-coding regions of mitochondrial and chloroplast DNA in plants. Molecular Ecology.

[PLT056C15] Devarumath RM, Hiremath SC, Rao SR, Kumar A, Sheelavanthmath SS (2005). Genome interrelationship in the genus *Eleusine* (Poaceae) as revealed through heteroploid crosses. Caryologia.

[PLT056C16] Devarumath RM, Sheelavanthmath SS, Hiremath SC (2010). Chromosome pairing analysis in interspecific hybrids among tetraploid species of *Eleusine* (Poaceae). Indian Journal of Genetics.

[PLT056C17] Dhingra A, Folta MK (2005). ASAP: amplification, sequencing & annotation of plastomes. BMC Genomics.

[PLT056C18] Dida MM, Devos KM, Kole C (2006). Finger millet. Genome mapping and molecular breeding in plants. Vol. 1, cereals and millets.

[PLT056C19] Dida MM, Srinivasachary RS, Bennetzen JL, Gale MD, Devos KM (2007). The genetic map of finger millet, *Eleusine coracana*. Theoretical and Applied Genetics.

[PLT056C20] Dida MM, Wanyera N, Dunn MLH, Bennetzen JL, Devos KM (2008). Population structure and diversity in finger millet (*Eleusine coracana*) germplasm. Tropical Plant Biology.

[PLT056C21] Duke JA, Wain KK (1981). Medicinal plants of the world. Computer index with more than 85000 entries.

[PLT056C22] Heinze B (2005). http://www.bfw.ac.at/200/1859.html.

[PLT056C23] Hilu KW (1988). Identification of the ‘A’ genome of finger millet using chloroplast DNA. Genetics.

[PLT056C24] Hilu KW (1995). Evolution of finger millet: evidence from random amplified polymorphic DNA. Genome.

[PLT056C25] Hilu KW, Alice LA (1999). Evolutionary implications of *MATK* indels in Poaceae. American Journal of Botany.

[PLT056C26] Hilu KW, deWet JMJ (1976). Domestication of *Eleusine coracana*. Economic Botany.

[PLT056C27] Hilu KW, Johnson JL (1992). Ribosomal DNA variation in finger millet and wild species of *Eleusine* (Poaceae). Theoretical and Applied Genetics.

[PLT056C28] Hilu KW, Johnson JL (1997). Systematics of *Eleusine* Gaertn. (Poaceae, Chloridoideae): chloroplast DNA and total evidence. Annals of the Missouri Botanical Garden.

[PLT056C29] Hilu KW, deWet JMJ, Seigler D (1978). Flavonoids and systematics of *Eleusine*. Biochemical Systematics and Ecology.

[PLT056C30] Hilu KW, Alice LA, Liang H (1999). Phylogeny of Poaceae inferred from matK sequences. Annals of the Missouri Botanical Garden.

[PLT056C31] Hiratsuka J, Shimada H, Whittier R, Ishibashi T, Sakamoto M, Kondo C, Honji Y, Sun CR, Meng BY, Li YQ, Kanno A, Nishizawa Y, Hirai A, Shinozaki K, Sugiura M (1989). The complete sequence of the rice (*Oryza sativa*) chloroplast genome: intermolecular recombination between distinct tRNA genes accounts for a major plastid DNA inversion during the evolution of cereals. Molecular and General Genetics.

[PLT056C32] Hiremath SC, Chennaveeraiah MS (1982). Cytogenetical studies in wild and cultivated species of *Eleusine* (Gramineae). Caryologia.

[PLT056C33] Hiremath SC, Salimath SS (1991). The quantitative nuclear DNA changes in *Eleusine* (Gramineae). Plant Systematics and Evolution.

[PLT056C34] Hiremath SC, Salimath SS (1992). The ‘A’ genome donor of *Eleusine coracana* (L.) Gaertn. (Gramineae). Theoretical and Applied Genetics.

[PLT056C35] Ibrahim RIH, Azuma J-I, Sakamoto M (2007). PCR–RFLP analysis of the whole chloroplast DNA from three cultivated species of cotton (*Gossypium* L.). Euphytica.

[PLT056C36] Ishii T, McCouch SR (2000). Microsatellites and microsynteny in the chloroplast genomes of *Oryza* and eight other Gramineae species. Theoretical and Applied Genetics.

[PLT056C37] Jena SN, Kumar S, Nair K (2009). Molecular phylogeny in Indian *Citrus* L. (Rutaceae) inferred through PCR–RFLP and trnL–trnF sequence data of chloroplast DNA. Scientia Horticulturae.

[PLT056C38] Kishimoto S, Aida R, Shibata M (2003). Identification of chloroplast DNA variations by PCR–RFLP analysis in *Dendranthema*. Journal of the Japanese Society of Horticultural Sciences.

[PLT056C39] Komatsu K, Zhu S, Fushimi H, Qui TK, Cai S, Kadota S (2001). Phylogenetic analysis based on 18S rRNA gene and *mat*K gene sequences of *Panax vietnamensis* and five related species. Planta Medica.

[PLT056C40] Lakshmi M, Senthilkumar P, Parani M, Jithesh MN, Parida A (2000). PCR–RFLP analysis of chloroplast gene regions in *Cajanus* (Leguminosae) and allied genera. Euphytica.

[PLT056C41] Liu Q, Triplett JK, Wen J, Peterson M (2011). Allotetraploid origin and divergence in *Eleusine* (Chloridoideae, Poaceae): evidence from low-copy nuclear gene phylogenies and a plastid gene chronogram. Annals of Botany.

[PLT056C42] Lye KA (1999). Nomenclature of finger millet (Poaceae). Lidia.

[PLT056C43] Murray MG, Thompson KH (1980). Rapid isolation of high molecular weight plant DNA. Nucleic Acids Research.

[PLT056C44] Mysore KS, Baird V (1997). Nuclear DNA content in species of *Eleusine* (Gramineae): a critical re-evaluation using laser flow cytometry. Plant Systematics and Evolution.

[PLT056C45] Nei M, Li WH (1979). Mathematical model for studying genetic variation in terms of restriction endonucleases. Proceedings of the National Academy of Sciences of the USA.

[PLT056C46] Neves SS, Kole C (2011). Eleusine. Wild crop relatives: genomic and breeding resources, millets and grasses.

[PLT056C47] Neves SS, Clark GS, Hilu KW, Baird WV (2005). Phylogeny of *Eleusine* (Poaceae: Chloridoideae) based on nuclear ITS and plastid *trnT*–*trnF* sequences. Molecular Phylogenetics and Evolution.

[PLT056C48] Nwakanma DC, Pillay M, Okoli E, Tenkouano A (2003). Sectional relationship in the genus *Musa* L. inferred from the PCR–RFLP of organelle DNA sequences. Theoretical and Applied Genetics.

[PLT056C49] Parani M, Lakshmi M, Ziegenhagen B, Fladung M, Senthikumar P, Parida A (2000). Molecular phylogeny of mangroves VII. PCR–RFLP of *trn*S–*psb*C and *rbc*L gene regions in 24 mangrove and mangrove-associated species. Theoretical and Applied Genetics.

[PLT056C50] Parani M, Rajesh K, Lakshmi M, Parducci L, Szmidt AE, Parida A (2001). Species identification in seven small millet species using polymerase chain reaction–restriction fragment length polymorphism of *trn*S–*psb*C gene region. Genome.

[PLT056C51] Phillips SM (1972). A survey of the *Eleusine* Gaertn. (Gramineae) in Africa. Kew Bulletin.

[PLT056C52] Phillips SM, Hedberg I, Edwards S (1995). Poaceae (Gramineae). Flora of Ethiopia and Eritrea.

[PLT056C53] Poczai P, Cseh A, Taller J, Symon DE (2011). Genetic diversity and relationships in *Solanum* subg. *Archaesolanum* (Solanaceae) based on RAPD and chloroplast PCR–RFLP analyses. Plant Systematics and Evolution.

[PLT056C54] Powell W, Morgante M, McDevitt R, Vendramin GG, Rafalski JA (1995a). Polymorphic simple sequence repeat regions in chloroplast genomes: applications to the population genetics of pines. Proceedings of the National Academy of Sciences of the USA.

[PLT056C55] Powell W, Morgante M, Andre C, McNicoll JW, Machray GC, Doyle JJ, Tingey SV, Rafalski JA (1995b). Hypervariable microsatellites provide a general source of polymorphic DNA markers for the chloroplast genome. Current Biology.

[PLT056C56] Powell W, Morgante M, Doyle JJ, McNicoll JW, Tingey SV, Rafalski JA (1996). Genepool variation in genus *Glycine* subgenus *soja* revealed by polymorphic nuclear and chloroplast microsatellites. Genetics.

[PLT056C57] Pradhan A, Nag SK, Patil SK (2010). Dietary management of finger millet (*Eleusine coracana* L. Gaerth) controls diabetes. Current Science.

[PLT056C58] Provan J, Corbett G, McNicoll JW, Powell W (1997). Chloroplast DNA variability in wild and cultivated rice (*Oryza* spp.) revealed by polymorphic chloroplast simple sequence repeats. Genome.

[PLT056C59] Provan J, Russel JR, Booth A, Powell W (1999). Polymorphic chloroplast simple sequence repeat primers for synthetic and population studies in the genus *Hordeum*. Molecular Ecology.

[PLT056C60] Provan J, Powell W, Hollingsworth PM (2001). Chloroplast microsatellites: new tools for studies in plant ecology and evolution. Trends in Ecology and Evolution.

[PLT056C61] Saitou N, Nei M (1987). The neighbour-joining method: a new method for reconstructing phylogenetic trees. Molecular Biology and Evolution.

[PLT056C62] Salimath SS (1990). Cytology and genome relations in some species of Eleusine and its allies.

[PLT056C63] Salimath SS, de Oliveira AC, Godwin ID, Bennetzen JL (1995a). Assessment of genome origins and genetic diversity in the genus *Eleusine* with DNA markers. Genome.

[PLT056C64] Salimath SS, Hiremath SC, Murthy HN (1995b). Genome differentiation patterns in diploid species of *Eleusine* (Poaceae). Hereditas.

[PLT056C65] Sehgal D, Rajpal VR, Raina SN (2008). Chloroplast DNA diversity reveals the contribution of the two wild species in the origin and evolution of diploid safflower (*Carthamus tinctorius* L.). Genome.

[PLT056C66] Shinozaki K, Ohme M, Tanaka M, Wakasugi T, Hayashida N, Matsubayashi T, Zaita N, Chunwongae J, Obokata J, Yamaguchi-Shinozaki K, Ohta C, Torazawa K, Meng BY, Sugita M, Deno H, Kamogashira T, Yamada K, Kusuda J, Takaiwa F, Kato A, Tohdoh N, Shimada H, Sugiura M (1986). The complete nucleotide sequence of tobacco chloroplast genome: its gene organization and expression. EMBO Journal.

[PLT056C67] Sneath PHA, Sokal RR (1973). Numerical taxonomy.

[PLT056C68] Swofford DL (2002). PAUP*: Phylogenetic analysis using parsimony (* and other methods), Version 4.

[PLT056C69] Taberlet P, Gielly L, Pautou G, Bouvet J (1991). Universal primers for amplification of three non-coding regions of chloroplast DNA. Plant Molecular Biology.

[PLT056C70] Tamura K, Peterson D, Peterson N, Stecher G, Nei M, Kumar S (2011). MEGA5: molecular evolutionary genetics analysis using maximum likelihood, evolutionary distance, and maximum parsimony methods. Molecular Biology and Evolution.

[PLT056C71] Thompson JD, Gibson TJ, Plewniak F, Mougin FJ, Higgins DG (1997). The Clustal X Windows interface: flexible strategies for multiple sequence alignment aided by quality analysis tools. Nucleic Acid Research.

[PLT056C72] Tsumura Y, Yoshimura K, Tomaru N, Ohba K (1995). Molecular phylogeny of conifers using RFLP analysis of PCR-amplified specific chloroplast genes. Theoretical and Applied Genetics.

[PLT056C73] Tsumura Y, Kawahara T, Wickneswari R, Yoshimura K (1996). Molecular phylogeny of Dipterocarpaceae in Southeast Asia using RFLP of PCR-amplified chloroplast genes. Theoretical and Applied Genetics.

[PLT056C74] Van Droogenbroeck B, Kyundt T, Maertens I, Romeij-Peeters E, Scheldeman X, Romero-Motochi JP, Van Damme P, Goetghebeur P, Gheysen G (2004). Phylogenetic analysis of highland papayas (*Vasconcellea*) and allied genera (Caricaeae) using PCR–RFLP. Theoretical and Applied Genetics.

[PLT056C75] Verma V (2009). Textbook of economic botany.

[PLT056C76] Weising K, Gardner RC (1999). A set of conserved PCR primers for the analysis of simple sequence repeat polymorphisms in chloroplast genomes of dicotyledonous angiosperms. Genome.

[PLT056C77] Werth CR, Hilu K, Langner CA, Baird WV (1993). Duplicated gene expression for isocitrate dehydrogenase and 6-phosphogluconate dehydrogenase in diploid species of *Eleusine* (Gramineae). American Journal of Botany.

[PLT056C78] Werth CR, Hilu KW, Langner CA (1994). Isozyme of *Eleusine* (Gramineae) and the origin of finger millet. American Journal of Botany.

[PLT056C79] Zhu S, Fushimi H, Cai S, Komatsu K (2003). Phylogenetic relationship in the genus *Panax*: inferred from chloroplast *trn*K gene and nuclear 18S rRNA gene sequences. Planta Medica.

