# Supplementary material for: Chloroplast genes as genetic markers for inferring patterns of change, maternal ancestry and phylogenetic relationships among Eleusine species
Source: AoB Plants. 2013 Dec 19;6:plt056. doi: 10.1093/aobpla/plt056 (PMC3924058; doi:10.1093/aobpla/plt056)
Supplement: Additional Information [file supp_6_plt056_index.html]

Chloroplast genes as genetic markers for inferring patterns of change, maternal ancestry and phylogenetic relationships among Eleusine species — Additional Information 

# Chloroplast genes as genetic markers for inferring patterns of change, maternal ancestry and phylogenetic relationships among *Eleusine* species

## Additional Information

Additional Information

**Files in this Data Supplement:**

- Additional Information - xls file
